# Unusual Focal Lung Uptake without CT Abnormality on a Bone Scan: What Might It Mean?

**DOI:** 10.3390/diagnostics12040934

**Published:** 2022-04-08

**Authors:** Marie-Claude Eberlé, Cyril Fersing, Sophie Guillemard, Diana Ilonca, Séverine Guiu, Emmanuel Deshayes

**Affiliations:** 1 Nuclear Medicine Department, Institut Régional du Cancer de Montpellier (ICM), University Montpellier, 34298 Montpellier, France; marie-claude.eberle@icm.unicancer.fr (M.-C.E.); sophie.guillemard@icm.unicancer.fr (S.G.); alina-diana.ilonca@icm.unicancer.fr (D.I.); emmanuel.deshayes@icm.unicancer.fr (E.D.); 2 IBMM, University of Montpellier, CNRS, ENSCM, 34293 Montpellier, France; 3 Medical Oncology Departement, Institut Régional du Cancer de Montpellier (ICM), University Montpellier, 34298 Montpellier, France; severine.guiu@icm.unicancer.fr; 4 Institut de Recherche en Cancérologie de Montpellier (IRCM), INSERM U1194, Institut Régional du Cancer de Montpellier (ICM), University Montpellier, 34298 Montpellier, France

**Keywords:** bone scintigraphy, extraosseous uptake, lung uptake, SPECT/CT

## Abstract

A 48-year-old woman was referred for a bone scan as an assessment of bone metastasis from breast cancer. Surprisingly, two hot spots of lung uptake were present in the left lung without any abnormality on CT slices. No history of pulmonary disease was observed. An optimized CT scan with fine slices performed the same day was strictly normal (without any micronodule). A lung ventilation/perfusion scintigraphy showed no significant perfusion defect. A follow-up bone scan performed eight months later was normal and without any lung uptake. After exclusion of the main etiologies described in the literature, such as amylosis, sarcoidosis, abscess, or hypercalcemia, radiotracer microembolism seems to be the most likely hypothesis in this patient.

**Figure 1:**
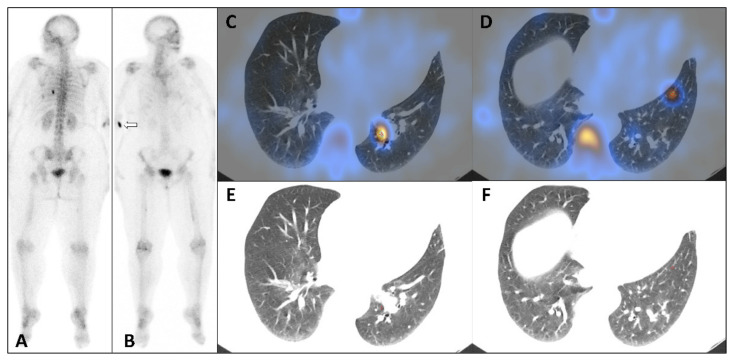
Whole body bone scan: posterior view (**A**), anterior view (**B**), axial SPECT-CT fused slices (**C**,**D**), correspondent CT slices (**E**,**F**). A 48-year-old woman was referred for a bone scan for assessment of bone metastasis from breast cancer. Posterior whole-body scan performed 2 h 30 min after IV injection of 825 MBq of ^99m^Tc-hydroxymethylene diphosphonate (^99m^Tc-HDP) highlighted two extra-osseous foci in projection of the 8th left rib (Figure 1A). Axial SPECT-CT fused slices showed an intense focal uptake in the left posterobasal lung segment close to proximal inferior lobe vessel (Figure 1C) and a less intense and more distal second focal uptake in the left anterobasal segment (Figure 1D). No abnormality was visible on correspondent CT slices (Figure 1E,F). Bone metastasis were limited to sternal body and left femoral diaphysis with mild osteoblastic activity. The patient had predominant liver metastasis, and carried out a combined treatment with trastuzumab, pertuzumab, letrozole, denosumab, calcium carbonate, and vitamin D3. The patient performed a contrast CT scan on the same day, thus, iobitridol was injected between ^99m^Tc-HDP intravenous injection and bone scan images acquisition. Fine helical slices showed no abnormality and especially no nodule or micronodule in the left lung. In order not to miss out a pulmonary embolism, ventilation/perfusion scintigraphy was performed five days later (Figure 2A,B), showing no significant perfusion defect. Eight months later, the follow-up bone scan showed no lung abnormality (Figure 2C,D), suggesting a non-pathological condition.

**Figure 2:**
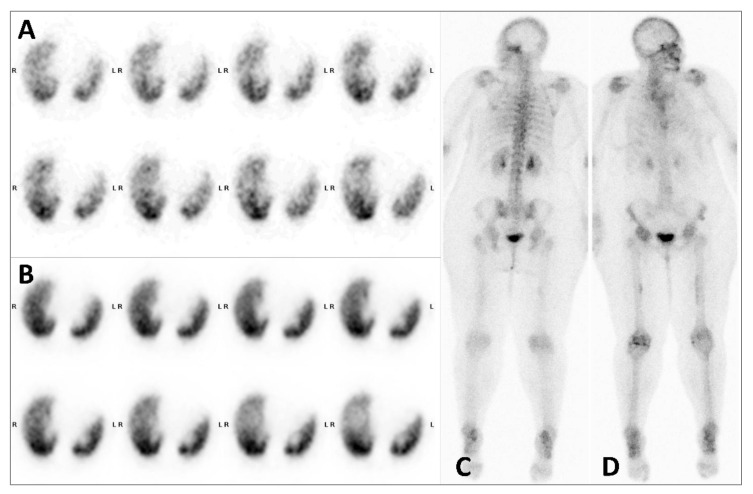
Subsequent scintigraphic investigations with ventilation/perfusion scintigraphy: ventilation axial SPECT slices (**A**), perfusion axial SPECT slices (**B**); and follow-up whole body bone scan: posterior view (**C**), anterior view (**D**). Extraosseous uptake on bone scan with ^99m^Tc-HDP or ^99m^Tc-methylene diphosphonate (^99m^Tc-MDP) have been well described, with great improved diagnosis accuracy in the era of SPECT/CT [1,2,3]. In particular, focal lung uptake can be due to several causes such as sarcoidosis, amylosis, lung metastasis, lung abscess, or hypercalcemia [4,5,6]. None of these conditions was present in our patient. Furthermore, unusual lung focal uptake with no morphological abnormality on CT scan were described with several radiotracers such as ^99m^Tc-dimercaptosuccinic acid (^99m^Tc-DMSA) [7], ^99m^Tc-sestamibi [8,9], ^68^Ga-DOTANOC [10], more recently ^68^Ga-PSMA-11 [11] and above all ^18^F-fluorodesoxiglucose (^18^FDG) with which lung hot spots could be present in up to 1.5/1000 exams [12,13]. Despite the great number of bone scan practiced worldwide since decades, this pattern seems extremely rare with bone-seeking tracers, described once with ^99m^Tc-MDP [14]. A paravenous radiotracer injection due to extravasation has been incriminated in a few cases [15]. Indeed, this was objectified in our patient (slight focal uptake near right elbow on anterior whole-body scan (Figure 1B, white arrow)). When performed, a new scintigraphy showing a normal pattern suggests that this cannot be due to pathological condition [12]. The conformity of the radiopharmaceutical preparation was not questionable (radiochemical purity > 97% and pH = 6, as recommended by the summary of products characteristics of the HDP cold kit). Although the patient dose was prepared 30 min before injection, neither instability nor significant interaction with the syringe were reported in the literature [16]. Furthermore, seven other patient syringes were prepared this day from the same multidose vial of ^99m^Tc-HDP and provided bone scans without such abnormality. In view of the clinic, and since the other causes already described seem to be excluded, the main causal hypothesis remains iatrogenic lung microemboli of the tracer, developed secondary to agglutination of ^99m^Tc-HDP with clotting elements that led to transient obstruction in tiny pulmonary arterioles.

## Data Availability

Not applicable.
